# Supplementary material for: MetaQTL: a package of new computational methods for the meta-analysis of QTL mapping experiments
Source: BMC Bioinformatics. 2007 Feb 8;8:49. doi: 10.1186/1471-2105-8-49 (PMC1808479; doi:10.1186/1471-2105-8-49)
Supplement: Additional File 7 — MetaQTL Package : jar file and tutorial. This Zip archive contains both the MetaQTL JAR file and the files of the tutorial. [file 1471-2105-8-49-S7.zip › org.inra.metaqtl/doc/org/metaqtl/bio/entity/BioEntityCollection.html]

BioEntityCollection


|  |  |  |
| --- | --- | --- |
| **Overview** \| **Package** \| **Class** \| **Use** \| **Tree** \| **Deprecated** \| **Index** \| **Help** | |  |
| **PREV CLASS**   **NEXT CLASS** | **FRAMES**    **NO FRAMES**     **All Classes** |  |
| SUMMARY: NESTED \| FIELD \| CONSTR \| METHOD | DETAIL: FIELD \| CONSTR \| METHOD |  |


---


## org.metaqtl.bio.entity Class BioEntityCollection

```
java.lang.Object
  java.util.AbstractCollection<E>
      java.util.AbstractList<E>
          java.util.ArrayList
              org.metaqtl.bio.entity.BioEntityCollection
```

**All Implemented Interfaces:**: java.io.Serializable, java.lang.Cloneable, java.lang.Iterable, java.util.Collection, java.util.List, java.util.RandomAccess, IBioAdaptable, IBioEntity

---

``` public class BioEntityCollection extends java.util.ArrayList implements IBioEntity, java.util.Collection ```

Class Description Here

**Author:**
:   Jean-Baptiste Veyrieras

**See Also:**: Serialized Form

---

| **Field Summary** | |
| --- | --- |
| `protected  java.lang.String` | `name` |
| `protected  IBioEntity` | `parent` |
| `protected  java.util.Properties` | `properties` |

**Fields inherited from class java.util.AbstractList**

`modCount`


**Constructor Summary**

`BioEntityCollection()`

`BioEntityCollection(int i)`


| **Method Summary** | |
| --- | --- |
| `IBioAdapter` | `getBioAdapter()` |
| `java.lang.String` | `getName()`             Returns the name of the biological entity |
| `IBioEntity` | `getParent()`             Returns the entity that handles this entity. |
| `java.util.Properties` | `getProperties()`             Gets the properties of the entity. |
| `int` | `getType()`             There are 2 main class of entities. |
| `void` | `setName(java.lang.String value)` |
| `void` | `setProperties(java.util.Properties defaultProps)`             Sets the properties of the entity. |

**Methods inherited from class java.util.ArrayList**

`add, add, addAll, addAll, clear, clone, contains, ensureCapacity, get, indexOf, isEmpty, lastIndexOf, remove, remove, removeRange, set, size, toArray, toArray, trimToSize`

**Methods inherited from class java.util.AbstractList**

`equals, hashCode, iterator, listIterator, listIterator, subList`

**Methods inherited from class java.util.AbstractCollection**

`containsAll, removeAll, retainAll, toString`

**Methods inherited from class java.lang.Object**

`finalize, getClass, notify, notifyAll, wait, wait, wait`

**Methods inherited from interface java.util.Collection**

`add, addAll, clear, contains, containsAll, equals, hashCode, isEmpty, iterator, remove, removeAll, retainAll, size, toArray, toArray`

**Methods inherited from interface java.util.List**

`containsAll, equals, hashCode, iterator, listIterator, listIterator, removeAll, retainAll, subList`

**Field Detail**

### name

```
protected java.lang.String name
```

---


### parent

```
protected IBioEntity parent
```

---


### properties

```
protected java.util.Properties properties
```


**Constructor Detail**

### BioEntityCollection

```
public BioEntityCollection()
```

---


### BioEntityCollection

```
public BioEntityCollection(int i)
```

**Parameters:**: `i` -


**Method Detail**

### getType

```
public int getType()
```

:   **Description copied from interface: `IBioEntity`**
:   There are 2 main class of entities. The first one deals with population
    biological entity, i.e population itself and individuals. The second class
    is a representation of microscopic biological entity from genome container
    to alleles.

    :   **Specified by:**: `getType` in interface `IBioEntity`

---


### getBioAdapter

```
public IBioAdapter getBioAdapter()
```

:   **Specified by:**: `getBioAdapter` in interface `IBioAdaptable`

---


### getName

```
public java.lang.String getName()
```

:   **Description copied from interface: `IBioEntity`**
:   Returns the name of the biological entity

    :   **Specified by:**: `getName` in interface `IBioEntity`

---


### setName

```
public void setName(java.lang.String value)
```

:   **Specified by:**: `setName` in interface `IBioEntity`

---


### getParent

```
public IBioEntity getParent()
```

:   **Description copied from interface: `IBioEntity`**
:   Returns the entity that handles this entity.

    :   **Specified by:**: `getParent` in interface `IBioEntity`

---


### getProperties

```
public java.util.Properties getProperties()
```

:   **Description copied from interface: `IBioEntity`**
:   Gets the properties of the entity.

    :   **Specified by:**: `getProperties` in interface `IBioEntity`

---


### setProperties

```
public void setProperties(java.util.Properties defaultProps)
```

:   **Description copied from interface: `IBioEntity`**
:   Sets the properties of the entity.

    :   **Specified by:**: `setProperties` in interface `IBioEntity`


---


|  |  |  |
| --- | --- | --- |
| **Overview** \| **Package** \| **Class** \| **Use** \| **Tree** \| **Deprecated** \| **Index** \| **Help** | |  |
| **PREV CLASS**   **NEXT CLASS** | **FRAMES**    **NO FRAMES**     **All Classes** |  |
| SUMMARY: NESTED \| FIELD \| CONSTR \| METHOD | DETAIL: FIELD \| CONSTR \| METHOD |  |


---
